# Armadillo domain of ARID1A directly interacts with DNA-PKcs to couple chromatin remodeling with nonhomologous end joining (NHEJ) pathway

**DOI:** 10.1093/nar/gkaf150

**Published:** 2025-03-13

**Authors:** Shin-ichiro Kanno, Takayasu Kobayashi, Reiko Watanabe, Akihiro Kurimasa, Kozo Tanaka, Akira Yasui, Ayako Ui

**Affiliations:** Division of Dynamic Proteome in Cancer and Aging, Department of Molecular Oncology, Institute of Development, Aging and Cancer, Tohoku University, Sendai, Miyagi 980-8575, Japan; Center for Animal and Gene Research, Tohoku University, Sendai, Miyagi 980-8575, Japan; Institute for Protein Research, Osaka University, 3-2 Yamadaoka, Suita, Osaka 565-0871, Japan; Division of Radiation Biology and Medicine, Faculty of Medicine, Tohoku Medical and Pharmaceutical University, Sendai, Miyagi 983-8536, Japan; Department of Molecular Oncology, Institute of Development, Aging and Cancer, Tohoku University, Sendai, Miyagi 980-8575, Japan; Division of Dynamic Proteome in Cancer and Aging, Department of Molecular Oncology, Institute of Development, Aging and Cancer, Tohoku University, Sendai, Miyagi 980-8575, Japan; Division of Dynamic Proteome in Cancer and Aging, Department of Molecular Oncology, Institute of Development, Aging and Cancer, Tohoku University, Sendai, Miyagi 980-8575, Japan

## Abstract

The SWI/SNF chromatin-remodeling complex that comprises multiple subunits orchestrates diverse cellular processes, including gene expression, DNA repair, and DNA replication, by sliding and releasing nucleosomes. AT-interacting domain-rich protein 1A (ARID1A) and ARID1B (ARID1A/B), a pivotal subunit, have significant relevance in cancer management because they are frequently mutated in a broad range of cancer types. To delineate the protein network involving ARID1A/B, we investigated the interactions of this with other proteins under physiological conditions. The ARID domain of ARID1A/B interacts with proteins involved in transcription and DNA/RNA metabolism. Several proteins are responsible for genome integrity maintenance, including DNA-dependent protein kinase catalytic subunit (DNA-PKcs), bound to the armadillo (ARM) domain of ARID1A/B. Introducing a knock-in mutation at the binding amino acid of DNA-PKcs in HCT116 cells reduced the autophosphorylation of DNA-PKcs and the recruitment of LIG4 in response to ionizing radiation. Our findings suggest that within the SWI/SNF complex, ARID1A couples DNA double-strand break repair processes with chromatin remodeling via the ARM domains to directly engage with DNA-PKcs to maintain genome stability.

## Graphical Abstract

**Graphical Abstract:**
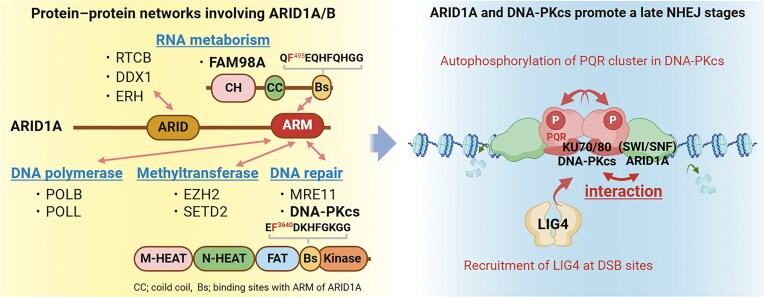

## Introduction

The SWitch/Sucrose Non-Fermentable (SWI/SNF) family complexes are ATP-dependent chromatin-remodeling factors that consist of a large complex with multiple subunits [1]. Along with histone modification enzymes, these factors play a crucial role in controlling gene expression by using the energy from ATP hydrolysis to move or remove nucleosomes, thereby altering chromatin structures. Recently, the diverse functionalities of the SWI/SNF family complexes have been revealed in transcriptional control, DNA damage repair, DNA replication, and chromosome segregation [1, 2].

The core subunits of the SWI/SNF complex are responsible for basic functions while accessory subunits determine variant properties. A recent comprehensive genome analyses showed that these subunits frequently undergo loss-of-function mutations in various cancer types [3]. Among these, the AT-interacting domain-rich protein 1A (ARID1A), an accessory subunit, contains mutations in ∼50% of ovarian clear cell carcinoma cases, as well as in gastric cancer (∼20%), bladder cancer (∼20%), liver cancer (∼15%), colorectal cancer, and breast cancer [4–7]. Mutations in ARID1B, the paralog of ARID1A, have also been identified in diverse tumor types such as ovarian, lung, and gastric cancers [8–10]. The ARID1A and ARID1B (ARID1A/B) proteins contain several important conserved domains: an N-terminal DNA-binding domain that selectively binds to AT-rich DNA sequences (ARID), a C-terminal β-catenin-like armadillo (ARM)-repeat fold involved in protein–protein interactions, and multiple copies of the LXXLL motif that stimulates the interaction of the protein with nuclear hormone receptors and regulates their transcriptional activity [11–14]. Furthermore, we previously found that ARID1A/B plays a crucial role in nonhomologous end joining (NHEJ) to maintain genome stability [2].

DNA-dependent protein kinase (DNA-PK) is a serine–threonine kinase consisting of a KU80/70 heterodimer and a catalytic subunit known as DNA-PKcs [15, 16] and plays a crucial role in repairing DNA double-strand breaks (DSBs). DSBs, typically caused by ionizing radiation (IR), are primarily repaired through NHEJ and homologous recombination, with DNA-PK being an essential factor in the NHEJ pathway [16, 17]. In NHEJ, the KU80/70 heterodimer bound to the ends of DSBs recruit DNA-PKcs to the damaged site and activates them. This activation facilitates the recruitment of NHEJ protein complexes required for repair to enable the rejoining of the damaged site [18]. DNA-PK was recently revealed to be involved in the repair of DNA damage repair and the regulation of various cellular functions, including DNA replication stress [19], gene expression, chromosome segregation [20], cell division, telomere capping [21], and activation of innate immunity [22]. In cancer cells, abnormalities in DNA-PK can contribute to cell proliferation, metastasis, tumor microenvironment establishment, and chromosome instability [23, 24].

Herein, we investigated the protein–protein interaction network involving ARID1A to understand the novel functions of ARID1A for genome stability. Consequently, we identified DNA-PKcs and the proteins involved in maintaining DNA/RNA homeostasis that act as caretakers of the genome as novel interactors with ARID1A. We found similar sequences between FAM98A and DNA-PKcs that interact with the ARM domains of ARID1A. Mutational disruption of the interaction with ARID1A/1B in one of these interacting proteins, the DNA-PK catalytic subunit (DNA-PKcs), reduced the autophosphorylation of DNA-PKcs and delayed the LIG4 recruitment in response to DSB. These results suggest that ARID1A within the SWI/SNF complex maintains genomic homeostasis through a newly identified protein–protein interaction.

## Materials and methods

### Cell culture

HEK293 cells were cultured in Dulbecco’s modified Eagle’s medium (Nissui) supplemented with 10% fetal bovine serum (FBS) under 5.0% CO_2_ at 37°C. HCT116 cells were maintained in RPMI1640 (Nissui) supplemented with 10% FBS.

### Identification of ARID1A-interacting proteins in HEK293 cells

Cells were homogenized in isotonic buffer (50 mM HEPES, pH 7.5, 0.2 M mannitol, 70 mM sucrose, 1 mM ethylenediaminetetraacetic acid (EDTA), 1 mM ethylene glycol tetraacetic acid (EGTA), 1.5 mM spermine, and 1.5 mM spermidine) containing a proteinase inhibitor cocktail and then centrifuged at 12 000 × *g* at 4°C for 10 min. Nuclear proteins were extracted with 50 mM HEPES, pH 7.5, 300 mM NaCl and 0.5% NP-40 containing DNase, RNase, and a proteinase inhibitor cocktail and then incubated with anti-ARID1A antibody (C-7, sc-373784, Santa Cruz) immobilized on protein A/G Magnetic Beads (Pierce) for 2 h at 4°C. Beads were washed three times with 50 mM HEPES, pH 7.5, 150 mM NaCl, and 0.5% NP-40, and bound proteins were eluted from the beads with 50 mM HEPES, pH 7.5, and 1.2 M NaCl for 30 min at 4°C. Eluted proteins were subjected to SDS–PAGE on a 5%–20% gradient gel and then stained with silver (Silver Stain MS Kit, Wako). Stained bands were excised from the gel and subjected to in-gel reduction with dithiothreitol, alkylation of the cysteine residues with iodoacetamide, and digestion with trypsin. Tryptic peptides were analyzed using a nanoflow high performance liquid chromatography–tandem mass spectrometry system.

### Immunoblot analysis

Immunoblot analysis was performed as described previously [25]. The following antibodies were used: anti-ARID1B (Santa Cruz, sc-32762), anti-VIRMA (25712-1-AP, Proteintech), anti-DDX1 (ab70252, abcam), anti-RTCB (A305-079-M, Betyl), anti-FAM98A (Bioss Antibodies, BS-11012R), anti-ERH (sc-373906, Santa Cruz), anti-DNA-PKcs (G-4, sc-5282, Santa Cruz), phospho-DNA-PKcs (Ser2056) (E9J4G, 68716, Cell Signaling Technology), anti-MRE11 (4895S, Cell Signaling Technology), anti-POLL (A301-641A, Betyl), anti-POLB (18003-1-AP, Proteintech), anti-SETD2 (A-3720-050, Epigentech), and EZH2 (21800-1-AP, Proteintech).

### Glutathione S-transferase (GST) pull-down assay

Fusion proteins of glutathione S-transferase (GST) with ARID1A mutants were overexpressed in *Escherichia coli* BL21(DE3). The GST pull-down assay was performed as described previously [26]. The BL21 expressing the ARID mutants was harvested and washed with phosphate-buffered saline (PBS) and lysed by sonication in GST lysis buffer (50 mM Tris–HCl, 0.3 M NaCl, 0.1% NP-40, and protease inhibitor). The lysate was clarified by centrifugation at 4°C, and the supernatant was transferred into a clean microcentrifuge tube. The supernatant was incubated with 50 μl of Glutathione Sepharose beads [50% (v/v); GE Healthcare] for 1 h at 4°C. The beads were washed four times with cold GST lysis buffer containing 0.3 M NaCl to wash out impurity proteins from BL21. Finally, the beads were washed with cold PBS. The ARID domain protein-bound beads were incubated in 293T cell nuclear extracts for 4 h in the presence of RNase A (10 mg/ml) and DNase I (10 mg/ml) at 4°C. Subsequently, the beads were washed thrice with washing buffer, added to SDS-sample buffer, and boiled for 5 min. Bound proteins were eluted in an SDS-sample buffer, resolved by SDS–PAGE (5%–20%), and processed for immunoblot analysis.

### Far-western analysis

Proteins were separated via SDS–PAGE (5%–20%) and transferred onto polyvinylidene difluoride membranes. The membrane was incubated in denaturing buffers from high to low concentrations of guanidine–HCl [3, 1.5, 0.75, and 0.375 M in Tris–HCl, pH7.5, 150 mM NaCl, and 0.01% Tween20 (TBS-T)] and then washed in TBS-T. After blocking with 0.3% skim milk in TBS-T for 1 h, the membrane was incubated with His–ARID1A–ARM overnight at 4°C. After washing with TBS-T, the membrane was probed with an anti-His-tag antibody (D291-3, MBL).

### Generation of DNA-PKcs knockin cell line

Homozygous mutations in F3640A knockin cells were generated using the CRISPR/Cas12a-mediated gene editing system (IDT). For cell nucleofection, Alt-R Cas12a crRNA (72 pmol) and Alt-R Cas12a Ultra protein (60 pmol) were preassembled for 15 min at room temperature, combined with donor oligonucleotide for homology-directed repair (HDR) (72 pmol) and ssDNA electroporation enhancer (43 pmol) (all reagents were purchased from IDT), and transfected into HCT116 cells using a Neon Transfection System (Thermo Fisher). Each transfection reaction was performed using 10^5^ cells per 10 μl of Neon Transfection tip at 1530 V, 20 ms, and 1 pulse setting. After transfection, cells were transferred to a culture plate containing 1 μM Alt-HDR enhancer v2 (IDT) and incubated for 5 days. Colonies were picked and transferred to 96-well plate. After genomic DNA extraction, the targeted region was polymerase chain reaction (PCR) amplified and then sequenced.

### Binding kinetics

Binding kinetics was analyzed using the Octet Red K2 (ForteBio, Menlo Park, CA, USA) and NTA biosensors. The His–ARID1A–ARM domain was immobilized on NTA biosensors. The wild-type GST–DNA–PKcs (3552–3691 aa), GST–DNA–PKcs F3640A mutation, GST FAM98A (479–519 aa), and GAT-POLB (7–53 aa) were diluted to various concentrations to measure the association and dissociation of the GST recombinant protein analyte to the His–ARID1A–ARM domain ligands (1914–2285 aa). Binding kinetics was measured using biolayer interferometry on the Octet. Association and dissociation curves were exported to ForteBio Data Analysis software (version 9.0) for analysis.

### Cell viability study

Cell viability was determined using PrestoBlue Cell Viability Reagents (Thermo Fisher) according to the manufacturer’s protocol.

### Immunofluorescence staining

Cells were fixed with 3% paraformaldehyde and 2% sucrose in PBS for 15 min, treated with 0.5% Triton X-100 in PBS (PBS-T) for 5 min, and blocked with 1% Block Ace (KAC) in PBS for 1 h at room temperature. Cells were incubated with anti-phospho-H2AX (05-636, Millipore), anti-53BP1 (A300-272A, Betyl), anti-FAM98A (Bioss Antibodies BS-11012R), anti-ARID1A (Sigma, HPA005456), and DNA-PKcs (G-4, sc-5282, Santa Cruz) antibodies for 1 h, washed three times with PBS-T, and incubated with Alexa Fluor 488 and Alexa Fluor 564 secondary antibody (Thermo Fisher) and 1 μM 4',6-diamidino-2-phenylindole (DAPI) for 1 h. Cells were washed three times with PBS-T and mounted in 1,4-diazabicyclo[2.2.2]octane (DABCO).

### Functional annotation of candidate genes

The Metascape (https://metascape.org/gp/index.html#/main/step1) database was used to explore the ontology terms of interacting proteins.

### Detection of NHEJ activities in cells

NHEJ activity was measured *in vivo* per Katsura *et al.*’s method [27]. The plasmid pEGFP-N1 was double-digested with EcoRI and BamHI and transfected into cells using Lipofectamine 2000. The medium was replaced 4 h later. Green fluorescent protein (GFP) fluorescence was measured 29 h after transfection using a GloMax Discover (Promega).

### Laser microirradiation and live imaging

Laser microirradiation and live imaging were performed [2, 28]. Before laser irradiation, HCT116 wild-type and F3640A mutant cells were transfected with GFP-fused plasmids, GFP–KU80, and GFP–LIG4, for 24 h. The irradiation dose was fixed at 1000 mW (500 scans at 1.6 mW/scan). At least four cells were irradiated in every experiment, and the live imaging of cells was performed for 200 s. To evaluate GFP-tagged protein accumulation, the mean intensity of each accumulated line was obtained after subtracting the background intensity in the irradiated cells within 200 s.

### Assay for site-specific homologous recombination activity

Assay for site-specific homologous recombination (HR) activity was performed [29]. After the transfection of ACTB–Cas9 and donor vectors, HCT116 wild-type and F3640A mutant cells were cultured for 72 h under 5.0% CO_2_ at 37°C. Genomic DNA was extracted, and quantitative PCR (qPCR) was performed. The relative quantity of the fusion gene was calculated.

### Immunoprecipitation

Immunoprecipitation was performed [30, 31]. Cells were extracted sequentially with NETN300 buffer [20 mM HEPES (pH 7.5), 300 mM NaCl, and 0.5% NP-40] containing Benzonase, a proteinase and phosphatase inhibitor cocktail (Roche), and incubated at 4°C for 30 min. The insoluble fraction was removed by centrifugation, whereas the soluble fraction was immunoprecipitated with anti-ARIDA1 or anti-DNA-PKcs antibody. Precipitated protein bands were analyzed by western blotting.

### Cell cycle analysis by flow cytometry

Cells were collected by trypsinization and centrifuged at 1000 rpm, washed in PBS, and fixed in ice-cold 70% ethanol with gentle vortexing. Cells were recentrifuged and washed with PBS, then incubated with PBS containing 200 μg/ml RNase A and 5 μg/ml PI. The cells were passed through a 40-μm cell strainer and analyzed by the flow cytometry (CytoFLEX LX, Beckman Coulter).

## Results

### Exploration of novel ARID1A-interacting proteins

**Figure 1.**
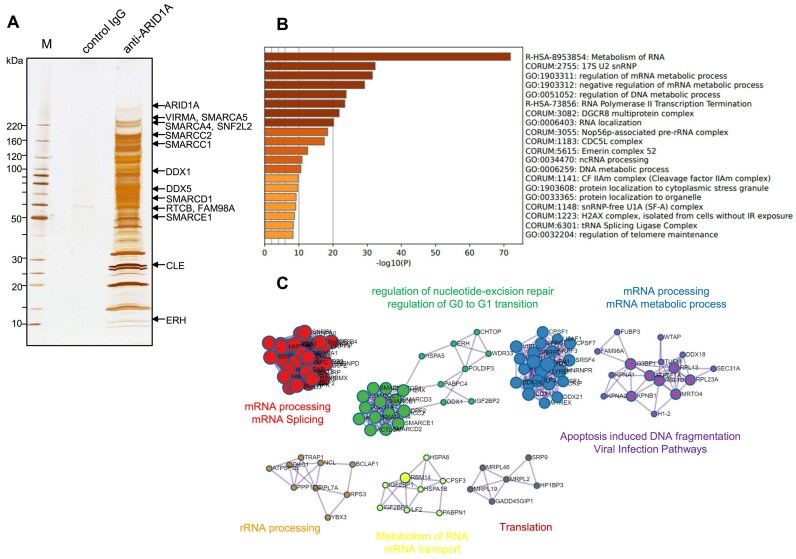
ARID1A interacts with various factors during transcription and RNA processing. (**A**) Silver-stained gel image of affinity-purified complexes of ARID1A; endogenous ARID1A was immunoprecipitated from nuclear extracts of HEK293 cells using specific antibodies, and bound proteins were separated by SDS–PAGE and silver-stained. Each band was analyzed using nanoflow HPLC-MS/MS. (**B**) GO (gene ontology) term enrichment analysis of ARID1A-interacting proteins using the Metascape database. (**C**) Protein–protein interaction network landscape of ARID1A-interacting proteins; functional descriptions of the corresponding components are also shown.

To identify proteins that interact with ARID1A under physiological conditions, a specific antibody was used to immunoprecipitate endogenous ARID1A and associated proteins from nuclear extracts of HEK293 cells. A total of 171 proteins were subsequently identified (Supplementary Fig. S1). As expected, subunits composing the SWI/SNF complex, such as SMARCA4 and actin-like 6A, were detected. However, a significant portion of the identified proteins were related to factors in DNA/RNA metabolism, including transcription, hnRNP proteins, small nuclear ribonucleoproteins, DEAD-box RNA helicases, and ribosomal proteins (Fig. 1A–C).

### Identification of the sequence required for FAM98A binding to the ARID1A ARM domain

**Figure 2.**
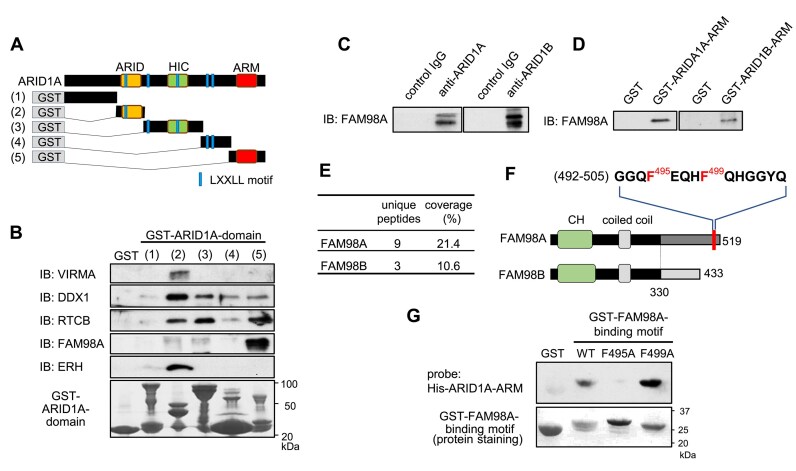
Identification of the sequence within FAM98A required for binding with ARID1A. (**A**) GST-fusion proteins with ARID1A mutants: (i) GST–ARID1A–N-terminal region (1–980 aa), (ii) GST–ARID1A–ARID domain (981–1140 aa), (iii) GST–ARID1A–HIC domain (1141–1641 aa), (iv) GST–ARID1A–C-terminal region (1634–1913 aa), (v) GST–ARID1A–ARM domain (1914–2285 aa). ARID: AT-rich interaction domain (1000–1120 aa), HIC1: hypermethylated in cancer 1 binding domain (1355–1451 aa), ARM: Armadillo domain (1974–2231 aa), and LXXLL: Leu-X-X-Leu-Leu sequence, a protein-recognition motif widely used in transcriptional regulation. (**B**) Mapping of the ARID1A region required for binding with interacting proteins; HEK293 nuclear extract was incubated with GST–ARID1A mutants, and interacting proteins were detected using the indicated antibodies. In addition, protein staining of GST–ARID1A mutants is shown (lower panel). IB, immunoblot. (**C**) FAM98A interacts with ARID1A and ARID1B. Endogenous ARID1A or ARID1B was immunoprecipitated with anti-ARID1A or -ARID1B, and FAM98A was probed by immunoblotting. (**D**) FAM98A interacts with ARID1A and ARID1B through their ARM domain. The nuclear extract was incubated with GST–ARID1A–ARM or ARID1B–ARM, and FAM98A was detected by immunoblot analysis. GST–ARID1B–ARM: ARM domain of ARID1B (aa 1861–2236). (**E**) Mass analysis data of FAM98A and FAM98B. (**F**) Comparison of the structures of FAM98A and FAM98B. FAM98A possesses a unique sequence in its C-terminal region. A sequence containing two adjacent phenylalanine residues is indicated. CH: calponin homology-like domain, black: highly conserved regions between FAM98A and FAM98B, dark gray: sequence-specific to FAM98A, and light gray: sequence-specific to FAM98B. (**G**) (Upper panel) Far-western blotting was used to detect the binding capacity of the wild-type, F495A mutant, or F499A mutant of the GST-fused C-terminal region of FAM98A (aa 479–519) to the His-tagged ARM domain of ARID1A. Protein staining of the GST-FAM98A C-terminal region is also shown (bottom panel).

To identify the specific ARID1A domains that interact with the novel interacting proteins, GST–ARID1A mutant proteins (Supplementary Fig. S2) were incubated with HEK293 nuclear extracts (Fig. 2A). Subsequently, VIRMA, DDX1, RTCB, FAM98A, and ERH were detected after GST pull-down with distinct interactions present among the various proteins (Fig. 2B). VIRMA and ERH bound explicitly to the GST–ARID1A mutant 2 (ARID domain), whereas FAM98A exhibited a specific interaction with the GST–ARID1A mutant 5 (ARM domain). DDX1 and RTCB interacted with multiple regions of ARID1A. We subsequently focused on FAM98A, which interacts with the ARM domain. FAM98A also interacts with endogenous ARID1A (Fig. 2C, left panel) and the paralog of ARID1A, ARID1B (Fig. 2C, right panel). FAM98 has several isoforms; therefore, these other isoforms may be detected in the immunoprecipitation blot (Fig. 2C) because, in addition to the direct interaction between the ARM domain of ARID1A and FAM98A, an indirect interaction may occur between full-length intracellular ARID1A and FAM98A through other cellular proteins. The interaction between FAM98A and ARID1B, as well as ARID1A, was mediated through the ARM domain of ARID1B (Fig. 2D, right panel).

FAM98A and its highly homologous ortholog, FAM98B were identified in the immunoprecipitation assay (Fig. 1A). However, the amount recovered for FAM98A was significantly higher than that for FAM98B (Fig. 2E). Therefore, we hypothesized that a specific structural feature present in FAM98A is involved in its interaction with ARID1A/B. Comparing the structures of FAM98A and FAM98B, we observed that although they share a high degree of similarity in the N-terminal region, they have distinct sequences in the C-terminal region (Fig. 2F). Although the ARID1A–ARM domain-binding region of FAM98A was not predicted to form α-helix by AlphaFold analysis, by using two different protein secondary structure prediction servers, this region is predicted to form α-helix (Supplementary Fig. S3A and B) and conserved in vertebrates (Supplementary Fig. S3C). We hypothesized that a sequence containing two adjacent phenylalanine residues in the C-terminal region of FAM98A is involved in its interaction with the ARM domain of ARID1A. Far-western blotting demonstrated that the C-terminal region of FAM98A (479–519 aa) directly bound the ARM domain of ARID1A (Fig. 2G lane 2). To investigate the significance of the phenylalanine residues in this interaction, we substituted alanine for Phe^495^ or Phe^499^ (Fig. 2G), of which only the replacement of Phe^495^ interrupted the interaction (Fig. 2G, lanes 3 and 4). The apparent molecular weight of the GST–FAM98A binding motif (F495A) is slightly different from WT and F499A mutants (Fig. 2G). The mobility of proteins on SDS–PAGE is influenced by the structure and properties of the polypeptide chain [32]. Therefore, structures exhibiting different mobility on SDS–PAGE may be associated with the binding ability of ARIDA to the ARM domain. These results demonstrate that FAM98A directly interacts with the ARM domain of ARID1A through the C-terminal sequence and that the phenylalanine residues in this region play a crucial role.

### Identification of novel binding proteins specific to the ARM domain of the ARID1A

**Figure 3.**
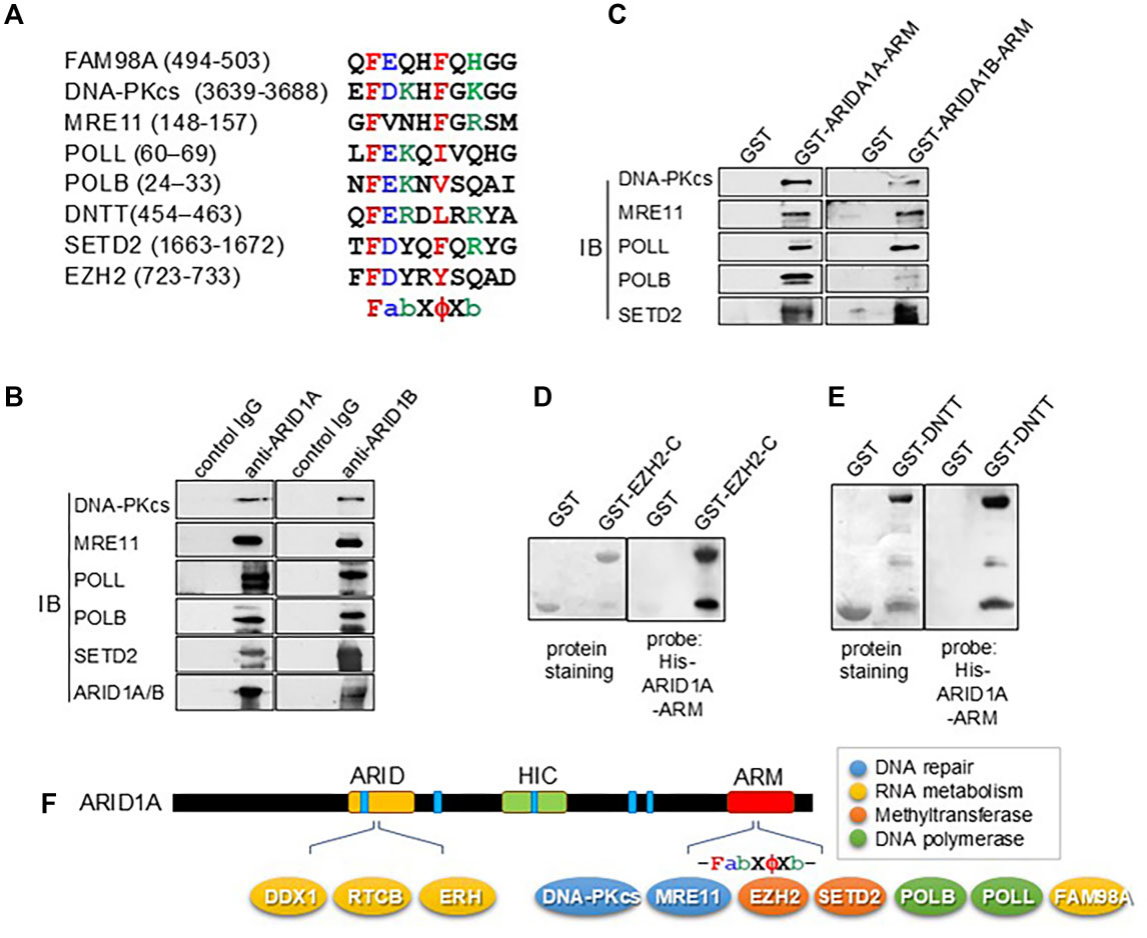
Identification of novel amino acid region involved in binding with the ARM domain of ARID1A. (**A**) Similar sequences of F(D/E)(K/R/H)XΦX(K/R/H) (X denotes any residue, whereas Φ represents a hydrophobic or aromatic residue) in DNA-PKcs, MRE11, POLL, POLB, DNTT, SETD2, and EZH2. (**B**) Proteins with the newly discovered region interact with ARID1A and ARID1B within the cell. The nuclear extract from HEK293 cells was immunoprecipitated with anti-ARID1A or -ARID1B antibody, and interacting proteins were probed by immunoblot analysis. (**C**and**D**) Proteins with the newly discovered region interact with ARID1A and ARID1B through their ARM domain. The nuclear extract was incubated with GST–ARID1A–ARM or ARID1B–ARM, and interacting proteins were probed with immunoblot analysis. (**C**) EZH2 and (**D**) DNTT interacted with the ARM domain of ARID1A using far-western blotting (right panels; His tag antibody). Protein-staining images of GST-fusion proteins (GST-EZH2-C: aa 598–746 and GST-DNTT: aa 1–509) are also shown (left panels). (**E**) Schematic of ARID1A and its interacting proteins.

We subsequently investigated whether sequences resembling those found in the C-terminal region of FAM98A, which are involved in the interaction with the ARM domain of ARID1A/1B, are present in proteins involved in regulating DNA repair, DNA recombination, and histone modification because we previously found that chromatin remodelers have important roles in DNA repair, HR and histone modification [2, 28, 30, 31, 33]. Consequently, we found sequences similar to that in FAM98A in several proteins, which included DNA-dependent protein kinase catalytic subunit (DNA-PKcs), which is activated in DSBs and plays a crucial role in NHEJ; meiotic recombination 11 homolog (MRE11), a nuclear protein involved in HR, telomere maintenance, and DSB repair; DNA polymerase λ (POLL), DNA polymerase β (POLB), and DNA nucleotidylexotransferase (DNTT), which are members of the X family of DNA polymerases that are involved in the process of DNA repair; SET domain containing 2 (SETD2), a histone lysine-specific methyltransferase involved in the initial response of HR repair; and enhancer of zeste 2 PRC2 subunit 2 (EZH2), a component of the polycomb repressive complex 2 (PRC2) that serves as a histone methyltransferase and plays an oncogenic role in various cancer types. Similar sequences are F(D/E)(K/R/H)XΦX(K/R/H) (X stands for any residue, and Φ represents a hydrophobic or aromatic residue) that were identified among these proteins (Fig. 3A).

DNA-PKcs, MRE11, POLL, POLB, and SETD2 were found to interact endogenously with ARID1A and its paralog, ARID1B (Fig. 3B). These proteins interact with ARID1A/B via their ARM domain (Fig. 3C). The interaction of EZH2 and DNTT with the ARM domain of ARID1A was confirmed *in vitro* using the far-western assay (Fig. 3D and F). We consequently found that the ARM domain of ARID1A builds novel interaction networks with factors involved in maintaining DNA/RNA homeostasis, including DNA repair, RNA metabolism, histone methyltransferase, and DNA polymerase (Fig. 3F).

### Physiological significance of the ARID1A ARM domain-binding site in DNA-PKcs

**Figure 4.**
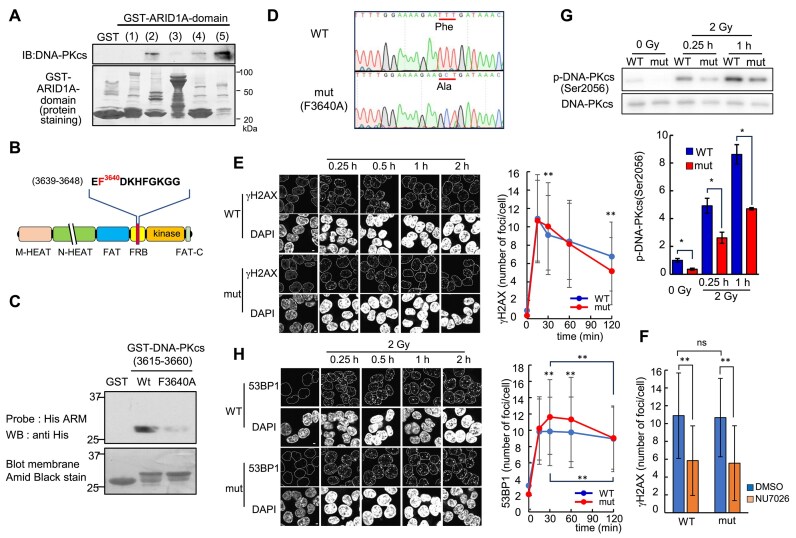
Generation of cells carrying DNA-PKcs knock-in mutation (F3640A) and its impact on cell properties. (**A**) Mapping of the ARID1A region required for binding with DNA-PKcs; HEK293 nuclear extract was incubated with GST–ARID1A mutants, and interacting proteins were detected using an antibody against DNA-PKcs (upper panel). GST-fusion proteins with ARID1A mutants are described in Fig. 2A. Protein staining of GST–ARID1A mutants is also shown (lower panel); IB, immunoblot. (**B**) Schematic of DNA-PKcs; phenylalanine residue that introduces substitution mutation is shown in red. HEAT: HEAT (Huntingtin, Elongation Factor 3, protein phosphatase 2A subunit, TOR) repeat-containing domain; FAT: a region of conservation among FRAP, ATM, and TRRAP; and FRB: FKBP12-rapamycin-binding. (**C**) Far-western blotting was used to detect the binding capacity of the wild-type or the F3640A mutant of the GST–DNA–PKcs ARID1A-binding region to the His-tagged ARM domain of ARID1A (upper panel). Protein staining of the GST–DNA–PKcs–ARID1A-binding domain is also shown (lower panel). (**D**) Generation of a homologous knock-in mutant strain of DNA-PKcs, in which the essential phenylalanine residue (Phe^3640^) for binding to the ARM domain of ARID1A is replaced with alanine. Sequencing results from genomic PCR that demonstrate the correct substitution are shown. (**E**) Wild-type (WT) or F3640A knock-in cells (mut) were unirradiated or irradiated with IR (2 Gy). After irradiation, the cells were subjected to immunofluorescent staining with antiphospho-H2AX (Ser139) antibody at the indicated time. DNA was counterstained with DAPI. The number of foci per cell counted by ImageJ is plotted. For each data point, 277–466 cells were analyzed. One of two independent experiments demonstrating similar results is presented. Data are presented as the mean ± SD. (**F**) WT or F3640A knock-in cells (mut) were pretreated with Dimethyl sulfoxide (DMSO) or NU7026 for 30 min and irradiated with IR (2 Gy). After 15 min, the cells were subjected to immunofluorescent staining with antiphospho-H2AX (Ser139) antibody. For each data point, 277–466 cells were analyzed. (**G**) WT or F3640A knock-in cells (mut) were exposed to IR (2 Gy) and harvested at the indicated time points for immunoblotting using regular (bottom panel) and phospho-specific (Ser2056, top panel) antibodies against DNA-PKcs. The results are expressed as the ratio of the value of WT obtained at 0 Gy. Data are presented as the mean ± SD, *n* = 3. *P < 0.05, **P < 0.01 (Student’s t-test). (**H**) Same as panel (E), except the antibody used is the anti-53BP1 antibody.

Despite the critical role that DNA-PKcs play in maintaining genome stability, their functional relationship with ARID1A/1B has not been previously elucidated. ARID1A partially colocalized with DNA-PKcs and FAM98A (Supplementary Fig. S4). We proceeded with further analysis of the interaction between these proteins. Initially, we used a GST pull-down to examine the binding specificity of ARID1A assay with GST-fusion proteins of various ARID1A domains (Fig. 2A shows the GST-mutant proteins used). DNA-PKcs exhibited weak binding to GST–ARID1A mutant 2 (ARID domain) and GST–ARID1A mutant 4 (C-terminal region). However, the most robust binding was observed with the ARM domain (GST–ARID1A mutant 5) (Fig. 4A). Next, we examined whether the sequence containing the putative binding site functions as an interacting domain between DNA-PKcs and ARID1A (Fig. 4B). Consequently, a GST-fusion protein of 45 amino acids of DNA-PKcs (F3640A) containing the binding site (3615–3660 aa) was prepared, and a far-western assay was performed using the His-tagged ARM domain of ARID1A as the bait. A direct interaction between the DNA-PKcs–binding region and the ARM domain of ARID1A was confirmed (Fig. 4C, lane 2). This interaction was disrupted when Phe3640 (F3640), which corresponds to the critical Phe^495^ in a binding region of FAM98A, was replaced with Ala (F3640A; Fig. 4C, lane 3). To confirm the interaction between F3640 of DNA-PKcs and ARID1A, we constructed another deletion mutant protein containing a longer amino acid region (3552–3691 aa) than the previous one to retain the intrinsic secondary structure around F3640. The mutant protein also disrupted the interaction between ARID1A and DNA-PKcs (Supplementary Fig. S5A). Additionally, we analyzed kinetic binding between ARID1A and its interactors, DNA-PKcs, FAM98A, and POLB, by BLI binding kinetics assay. Serial dilutions of recombinant wild-type DNA-PKcs, FAM98A, and POLB were bound to His–ARID1A–ARM, but the DNA-PKcs F3640A mutation decreased its binding (Supplementary Fig. S5B). These results suggest that DNA-PKcs directly interact with the ARM domain of ARID1A through a sequence containing a newly identified site.

To elucidate the physiological significance of the novel ARM domain-binding site in DNA-PKcs, CRISPR/Cas12a was used to create a mutant strain of HC116 cells (derived from human colon cancer) where the Phe^3640^ residue in DNA-PKcs was replaced with an Ala residue (Fig. 4D). To examine the influence of the mutation on the interaction between DNA-PKcs and ARID1A in the cells, we performed immunoprecipitation using a DNA-PKcs antibody in HCT116 wild-type and F3640A mutant cells. Before IR treatment, the DNA-PKcs F3640A mutation exhibited a decreased interaction with KU80 and ARID1A (Supplementary Fig. S5C), compared with wild-type DNA-PKcs, indicating that in the absence of DSBs, ARID1A may be involved in the interaction between KU70/80 and DNA-PKcs. IR treatment mitigated the reduced interaction with KU80 and ARID1A induced by the F3640 mutation (Supplementary Fig. S5D). Therefore, in the presence of DSBs, either this single mutation alone does not affect the interaction between full-length DNA-PKcs and ARID1A, or the two interact with each other via other NHEJ factors at DSB sites.

The generation of DSBs induces rapid phosphorylation of nearby H2AX, which triggers the recruitment of DNA damage repair proteins. DNA-PKcs is activated at DSB ends by the KU80/70 heterodimer and phosphorylates H2AX [34, 35]. In wild-type cells, the number of γH2AX foci induced by IR peaked 15 min after irradiation and gradually declined (Fig. 4E). DNA-PKcs is involved in this phosphorylation, as shown by the inhibition of γH2AX foci formation following treatment with NU7026, a DNA-PKcs kinase inhibitor (Fig. 4F). The formation of γH2AX foci induced by IR occurred in F3640A mutant cells to the same extent as that in wild-type cells, suggesting that the F3640A mutation does not affect the H2AX phosphorylation capability of DNA-PKcs (Fig. 4F). We also found that 53BP1 recruitment, which depends on γH2AX formation after DSB induction, was not affected by the F3640A mutation, whereas inhibition of the kinase activity of DNA-PKcs treated with NU7620 reduced it (Supplementary Fig. S6A). These results also suggested that the F3640A mutation did not affect the activation of DNA-PKcs kinase.

In addition to the DSB signal transduction initiated by H2AX phosphorylation, DNA-PKcs plays a crucial role in NHEJ, the primary mechanism of DSB repair. Interestingly, the F3604A mutation had little influence on IR sensitivity, cell cycle progression, and NHEJ activity [27] (Supplementary Figs S6B, C, and S7). DNA-PKcs possess two autophosphorylation clusters known as the ABCDE and PQR clusters [36–39]. PQR cluster mutations do not show significant sensitivity to IR compared with ABCDE cluster mutations. Furthermore, the P and the QR site mutations do not affect IR sensitivity [36]. In addition, while ABCDE cluster mutations inhibit HR, PQR cluster mutations promote it by facilitating DNA end processing to overcome DSBs [36] because PQR autophosphorylation potentially disrupts the Artemis association with DNA-PKcs [36, 38]. This is why PQR cluster mutations do not show significant sensitivity to IR [36]. As well as PQR cluster mutants, we found that the F3640A mutant was proficient in HR (Supplementary Fig. S8) [36], indicating that the F3640A phenotype is similar to those of PQR mutations. Therefore, we measured the phosphorylation level of the Ser^2056^ site within the PQR cluster to investigate whether the interaction with ARID1A is involved in the function of DNA-PKcs in NHEJ. The F3640A mutant exhibited lower IR-dependent autophosphorylation levels at Ser^2056^ than those in the wild-type (Fig. 4G), and emphasizing the contrast of WB data in Fig. 4G reveals that all lanes have signals (Supplementary Fig. S9A). This result suggested a delay in the NHEJ process in the mutant.

**Figure 5.**
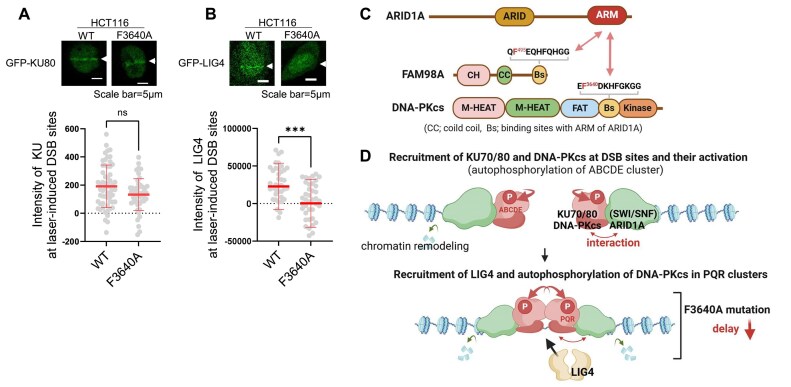
Interaction between DNA-PKcs and ARID1A facilitates late-stage NHEJ processes. (**A**and**B**) HCT116 WT and F3640A mutant cells were transfected with GFP-fused plasmids, GFP-KU80 and GFP-LIG4, for 24 h before irradiation. Following laser microirradiation, live-cell imaging was performed, capturing images every 10 s for 200 s. Representative images (upper panel) and quantification of signal intensity over 200 s (lower panel) of GFP-KU80 (A) and GFP-LIG4 (B) at DSB sites are shown. Scale bar: 10 μm. Statistical significance (Mann–Whitney U-test): ns, nonsignificant; ***P < 0.001. (**C**) ARM domain of ARID1A interacts with FAM98A and DNA-PKcs via their novel binding sites. (**D**) Proposed model of ARID1A in DSB repair. The DNA-PKcs–ARID1A interaction facilitates late-stage NHEJ processes, including PQR autophosphorylation and LIG4 recruitment, following KU70/80 and DNA-PKcs recruitment and activation at DSB sites. Through its interaction with DNA-PKcs, ARID1A, as part of the SWI/SNF complex, may promote nucleosome remodeling around DSB sites to support NHEJ. This interaction is proposed to induce conformational changes between DNA ends and the DNA-PKcs/KU70/80 complex, altering the autophosphorylation status of DNA-PKcs and enabling the recruitment of additional NHEJ factors, such as LIG4, during late-stage NHEJ.

Next, we determined the NHEJ processes affected by the F3640A mutation. The F3640 mutation did not affect the recruitment of KU80 at DSB sites (Fig. 5A), in addition to the phosphorylation of H2AX (Fig. 4F). DSBs are possibly repaired by NHEJ in the first 1–2 h after IR treatment, after which NHEJ is gradually replaced by HR [40]. Compared with the wild-type cells, the F3640A mutant cells showed high retention of γH2AX, a marker of DSBs, and 53BP1, a promoting factor of NHEJ, within 120 min after IR treatment, suggesting a delayed NHEJ process in the mutant strain (Fig. 4E and H). The summary of the number of cells analyzed in Fig. 4E, F, and H is added in Supplementary Fig. S9B. Furthermore, the F3640A mutant inhibited the recruitment of LIG4 at DSB sites (Fig. 5B), which occurs in late NHEJ stages. The mutant strain exhibited slower growth than the wild-type strain (Supplementary Fig. S9C), suggesting that a delay of the NHEJ pathway may affect cell growth. These results indicate that the interaction between DNA-PKcs and ARID1A promotes the processes in late NHEJ stages, including PQR autophosphorylation and LIG4 recruitment, after KU70/80 and DNA-PKcs are recruited and activated at DSB sites.

## Discussion

### Novel protein–protein networks involving ARID1A and ARID1B (ARID1A/B) in SWI/SNF chromatin-remodeling complex

The ARM repeat is a protein–protein interaction domain comprising multiple units, each containing 42 amino acids [41]. Herein, we found that proteins involved in regulating RNA/DNA homeostasis, including RNA processing, DSB repair, and histone modification, interact with the ARM domains of ARID1A/B through a novel binding site (Fig. 5C). Although SWI/SNF containing ARID1A/B is known to regulate transcription [8], we additionally found that the ARM domain of ARID1A interacts with two distinct histone methyltransferases, SETD2 and EZH2, to induce methylation of H3K36me3 and H3K27me3 to facilitate transcriptional activation and repression, respectively [42, 43]. Thus, ARID1A/B in the SWI/SNF complex may regulate transcription by directly interacting with factors with opposing transcriptional activities. Previously, Li *et al.* revealed that ARID1A suppresses the inhibitory effects of the histone methyltransferase EZH2 on the expression of genes encoding T-cell–inducing chemokines, thereby promoting the accumulation of T cells in the vicinity of cancer cells and ultimately suppressing cancer [44]. Their study highlighted the importance of the interaction between EZH2 and the ARM domain of ARID1A in exerting this effect. However, they did not identify the specific region in EZH2 involved in the interaction, and the site we elucidated in this study may play a role in this interaction. In addition, the SWI/SNF complex has been previously found to colocalize with RNA-dependent bodies such as nuclear stress bodies and paraspeckles and was essential for organizing the protein–protein interaction network within paraspeckles [45, 46]. Recently, the SWI/SNF complex containing ARID1B, rather than ARID1A, was found to preferentially interact with paraspeckle proteins (PSPs); furthermore, ARID1B was found to mediate the interaction of the SWI/SNF complex with the PSPs and engagement of paraspeckles to chromatin-associated proteins [47]. Our demonstration that FAM98A, a paraspeckle component, directly interacts with the ARM domain of ARID1A/B may help elucidate the association between ARID1A/B and paraspeckles (Fig. 5C). Reportedly, although DNA-PKcs and KU70/80 function in pre-rRNA biogenesis independent from DSB repair [48], ARID1A interacts with proteins involved in RNA processing and metabolism (Fig. 1) and is involved in the DNA-PKcs and KU70/80 interaction in the absence of DSBs (Supplementary Fig. S5). Therefore, there is a possibility that ARID1A forms another complex with DNA-PKs and KU70/80 and is involved in RNA processing and metabolism independent of DSB repair.

### Novel functions of ARID1A in NHEJ pathway via DNA-PKcs

The SWI/SNF complex serves additional crucial roles in the regulation of DSB repair to maintain genome stability. Consequently, beyond the transcriptional function of SWI/SNF, the involvement of this complex in DSB repair has been proposed to correlate with its tumor suppression activity [8]. Previous studies have demonstrated that the ATPase catalytic subunit of the SWI/SNF chromatin-remodeling complex containing ARID1A is crucial for recruiting KU70/80 to DSBs [2], suggesting that SWI/SNF-mediated chromatin remodeling around DSBs enables KU70/80 recruitment to DNA. In this study, we found that the ARID1A ARM domain directly interacts with the conserved binding sequence of DNA-PKcs, FAM98A, and POLB (Fig. 2A and Supplementary Fig. S5B). Furthermore, using GST pull-down assays with two distinct GST–DNA-PKcs constructs (Fig. 4C and Supplementary Fig. S5A) and a BLI binding kinetics assay (Supplementary Fig. S5B), we demonstrated that the F3640 residue within the binding sequence of DNA-PKcs is essential for its interaction with the ARID1A ARM domain (Fig. 5C). Furthermore, we showed that F3640A mutant impairs the interaction with ARID1A (Supplementary Fig. S5C), PQR autophosphorylation (Fig. 4G), the repair of DSBs (Fig. 4E and H), and LIG4 recruitment (Fig. 5B), indicating that the interaction between ARID1A and DNA-PKcs is necessary for the efficient progression of NHEJ. Previous reports suggested that during NHEJ, the activated DNA-PKcs–KU70/80 complex on the DSB end undergoes autophosphorylation in the ABCDE clusters (in *cis*), leading to the conformational change of the complex and the recruitment of other NHEJ factors, including Artemis, XRCC4, and LIG4, at the DSB sites [35, 49]. Subsequently, the sequential autophosphorylation in the PQR (in *trans*) clusters may disrupt the interaction between Artemis and DNA-PKcs and dissociate them [36, 49]. Thus, PQR cluster mutations prolong the binding of Artemis to DNA-PKcs, leading to excessive DNA end processing and promoting other DSB repair, including the HR pathway and survival of DSBs [36, 38, 50]. The F3640A mutation did not affect IR sensitivity, phosphorylation of H2AX at DSB sites, and recruitment of KU80 to DSB sites (Figs 4 and 5, and Supplementary Fig. S6). However, the F3640A mutation showed high retention of γH2AX and 53BP1 at DSB sites, autophosphorylation of Ser^2056^ in the PQR cluster, and LIG4 recruitment (Figs 4 and 5) indicating that the mutation delays the process in late NHEJ stages (Fig. 5D, lower step). Based on these findings, we propose the following models: through the interaction between ARID1A and DNA-PKcs, SWI/SNF may promote nucleosome remodeling around DSB sites to encourage NHEJ processes, leading to conformational changes between DNA ends and DNA-PKcs/KU70/80 complex which alter the autophosphorylation status of DNA-PKcs, and the recruitment of other NHEJ factors, including LIG4, in late NHEJ stages (Fig. 5D). Our results indicate the novel roles of the functional interaction between ARID1A in the SWI/SNF complex and the KU80 and DNA-PKcs complexes in DSB repair.

## Supplementary Material

gkaf150_Supplemental_Files

## Data Availability

The data and supporting materials are available within the article and from its online Supplementary Data.
