# Supplementary material for: Armadillo domain of ARID1A directly interacts with DNA-PKcs to couple chromatin remodeling with nonhomologous end joining (NHEJ) pathway
Source: Nucleic Acids Res. 2025 Mar 13;53(5):gkaf150. doi: 10.1093/nar/gkaf150 (PMC11904782; doi:10.1093/nar/gkaf150)
Supplement: gkaf150_Supplemental_Files [file gkaf150_supplemental_files.zip › Revised Supplementary Figures_Kanno et al.pdf]

Supplementary Figure S1

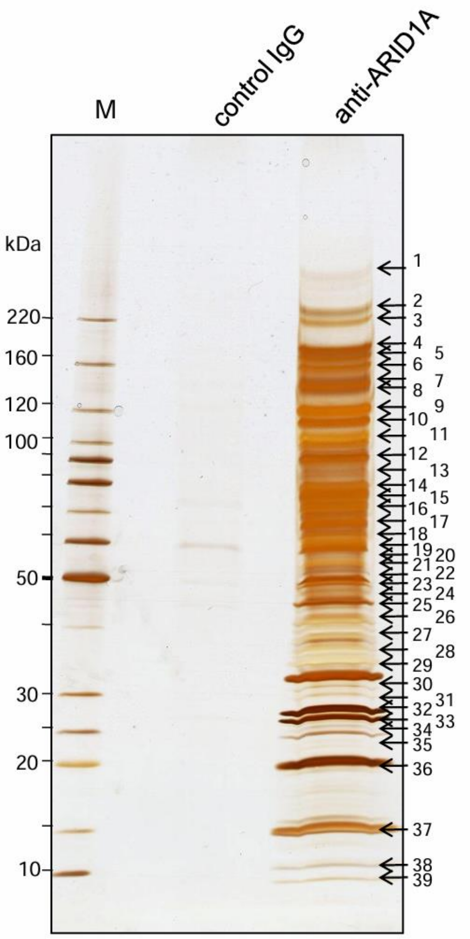

1: ARID1A, SNRPD1, MKI67

2: VIRMA, SMARCA5, SNRPD1, CDK18

3: BRG1, SNF2L2, SNRPD1

4: SMARCC2, CPSF1, ARID1A, SCAF4

5: SMARCC2, SMARCC1, SCAF4, ARID1A, WDR33

6: SMARCC1, U2SURP, SFRS4

7: DHX9, , U2SURP, SEC31L1, SCAF4, SMARCC2

8: SF3B3, SMARCC2, U2SURP, THRAP3, SCAF4, SNRPD1

9: HNRNPU, SKIV2L2, THRA, EIF2S2

10: ILF3, HNRNPU, SFPQ, NCL

11: SFPQ, WBP11, NCL, PRPSAP2

12: DDX1, KPNB1, HNRNPU, SFPQ, DHX15, ILF3, P3H1, MED16, DDX21, APPL1, KHSRP, PRP3, SNRPD1, EWSR1, MAPKAPK2, BCLAF1

13: SEC23A, HNRNPU, PLOD1, KHSRP, HNRNPR, DDX18, , EWSR1, DDX17, RANGAP1, DnaJ, BCLAF1, DDX1, PFPQ, CKI

14: HSPA5 , PABPC4 , DDX17, HNRNPU, HP1BP3, DDX3X, CPSF3, EWSR1

15: TRAP1, PABPN1, PABPC4, RBM14, HSPA8, HNRNPQ

16: HSPA1B, IGF2BP3, HNRNPR, DDX17, FUS, ORC2, SF1, IGF2BP2, SDHA, HNRNPU, HNRNPL, IGF2BPI, SNRPD1, EWSR1, LRWD1, NXF1

17: DDX5, IGF2BP1, HNRNPL, RSL1D1, HNRNPQ, FUS, USP39, EWSR1, G3BP1, KHDRBS1, IGF2BP3, NCOA4, IGF2BP2, PHF10

18: HNRNPL, HNRNPK, PSPC1, PHF10, U2AF, SF3B2, KPNA1, FUS, G3BP1, DDX5, GLTSCR2, SNRNP70, CARM1, FUBP3,

19: SMARCD1, HSPD1, SMARCD2, NONO, SMARCD3(BAF60C), U2AF2

20: NONO, FAM98A, SMARCD1, RTCB, CPSF7, YBX3, DKC1, PRPF4

21: RTCB, HNRNPK, CPCF7, NONO, KPNA2, FAM98A, WTAP

22: SMARCE1, RUVBL1, ATP5A, RBM17, RBBP7

23: SMARCE1, PD1A5, ATP5B, RUVBL2, RBM17, HNRNDL, HNRNPH

24: POLDIP3, ACTL6A, YBX1, BAF45D, NONO, SF3B4, FAM98B, SSB, SMARCE1, PDIA6

25: SMARCB1, HNRNPD0, TUFM, NF45, HNRNPA/B, FAM98B, HNRNPG

26: NF45, ACTB, ACTN2, HNRNPG, SMARCB1

27: HNRNPA3, HNRNPC, HNRNPA/B

28: HNRNPAA2/B1, HNRNPC1/C2, HNRNPA1, NPM1, HNRNPA0, HNRPCL1, CENP-V, PPP1CC

29: HNRNPA1, HNRNPA2/B1, HNRNPA0, HNRNPA3, ERCC3

30: SNRPA, C1QBP, HISTH1C, SNRPB2

31: RPL7a, MRTO4, RPS3, SRSF1, HIST1H1C, RQCD1, MRPL19, CHTOP, YWHAE,THOC4

32: SNRPA1, MRPL46, MRPL2, MRPL19, ASHWIN, CHTOP

33: C14ORF166, SNRPB, SNRPB2, RPL13

34: NUDT21, C14ORF166, SNRPB, PRDX3

35: PRDX3, ATP5O, PRDX3, NUDT21, GADD45GIP1, H2AX, Ub

36: SNRPC, ROL11, RPL23a, PRDX3

37: SNRPD3, SNRPD2, SNRPD1, HIST1H2BA,

38: SNRPE, SNRPF1, ERH, SRP9

39: SNRPG, SNRPE

Supplementary Figure S1. Identification of ARID1A-interacting proteins in HEK293 cells.

Endogenous ARID1A was immunoprecipitated from nuclear extracts of HEK293 cells using specific antibodies. Bound proteins were separated by SDS-PAGE, and the gel was silver-stained. Each band was analyzed using a nanoflow HPLC-MS/MS. Analysis of high-molecular-weight proteins is presented.

Supplementary Figure S2

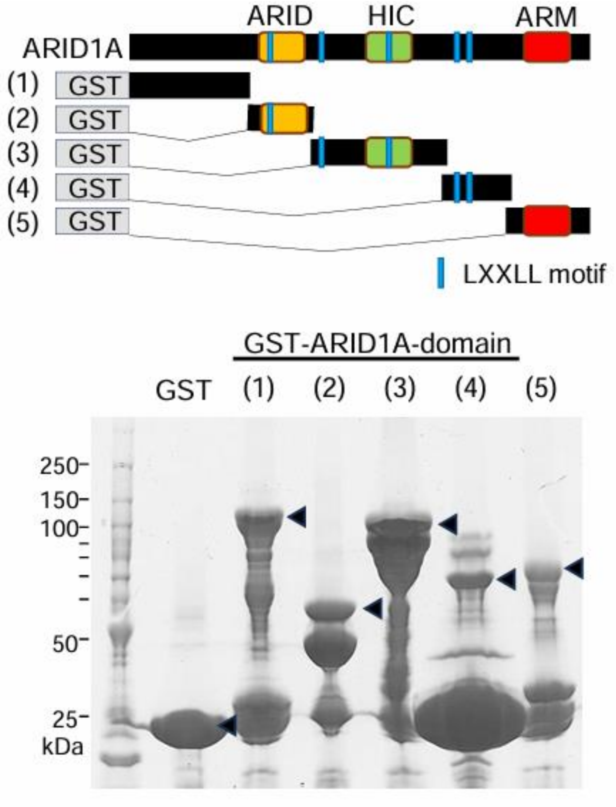

Supplementary Figure S2. GST-ARID1A mutant proteins.

(upper panel) GST fusion proteins with ARID1A mutants; (1): GST-ARID1A-N-terminal region (aa 1–980), (2): GST-ARID1A-ARID domain (aa 981–1,140), (3): GST-ARID1A-HIC domain (aa 1,141–1,641), (4): GST-ARID1A-C-terminal region (aa 1,634–1,913), and (5): GST-ARID1A-ARM domain (aa 1,914–2,285). ARID: AT-rich interaction domain (aa 1,000–1,120), HIC1: hypermethylated in cancer 1 binding domain (aa 1,355–1,451), ARM: Armadillo domain (aa 1,974–2,231), and LXXLL: Leu-X-X-Leu-Leu sequence—a protein-recognition motif widely used in transcriptional regulation. **(Lower panel)** GST-ARID1A mutant proteins are purified from *Escherichia coli* BL21.

Supplementary Figure S3

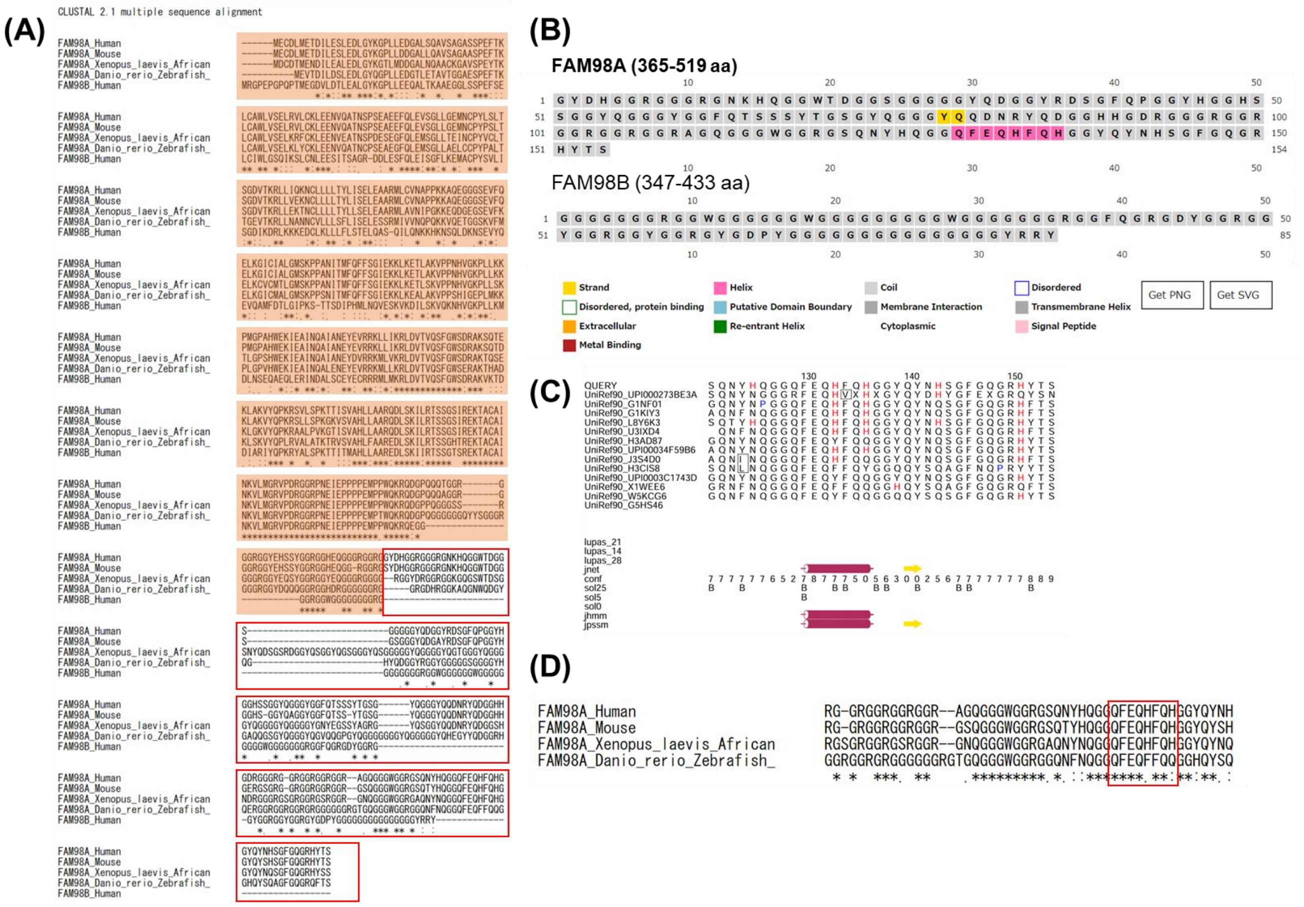

Supplementary Figure S3. Similar sequences in FAM98A and other interacting proteins to ARID1A.

(A) Comparing a complete sequence alignment of FAM98A and FAM98B (Multiple Sequence Alignment - CLUSTALW), we observed that although they share a high degree of similarity in the N-terminal region (orange), they have distinct sequences in the C-terminal region (red box).

(B-C) Prediction of the secondary structure of the C-terminal region in FAM98A and FAM98B (the sequences marked in red in (A)); FAM98A is speculated to have a C-terminal α-helix (sequence marked pink); secondary structure was predicted usingPSIPRED Workbench (http://bioinf.cs.ucl.ac.uk/psipred/) **(B)** and Jpred 4

(https://www.compbio.dundee.ac.uk/jpred4/) **(C).**

(D) α-helix in (B) and (C) is conserved in vertebrates (red box) (Multiple Sequence Alignment - CLUSTALW).

Supplementary Figure S4

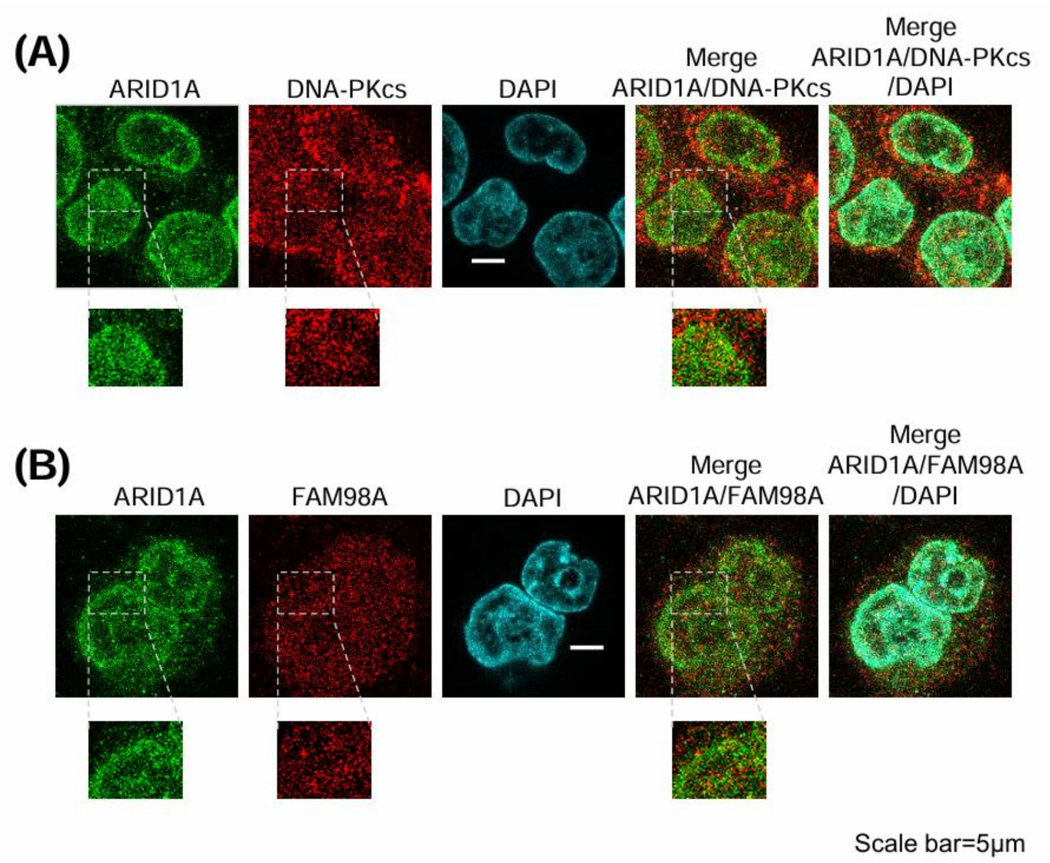

Supplementary Figure S4. Colocalization of ARID1A with DNA-PKcs or FA98A in nuclei.

HCT116 cells grown on lass bottom dishes were fixed and permeabilized. Immunofluorescence was performed in the cells using ARID1A, (A) DNA-PKcs, and (B) FAM98A antibodies. ARID1A partially colocalized with DNA-PKcs or FAM98A in nuclei.

Supplementary Figure S5

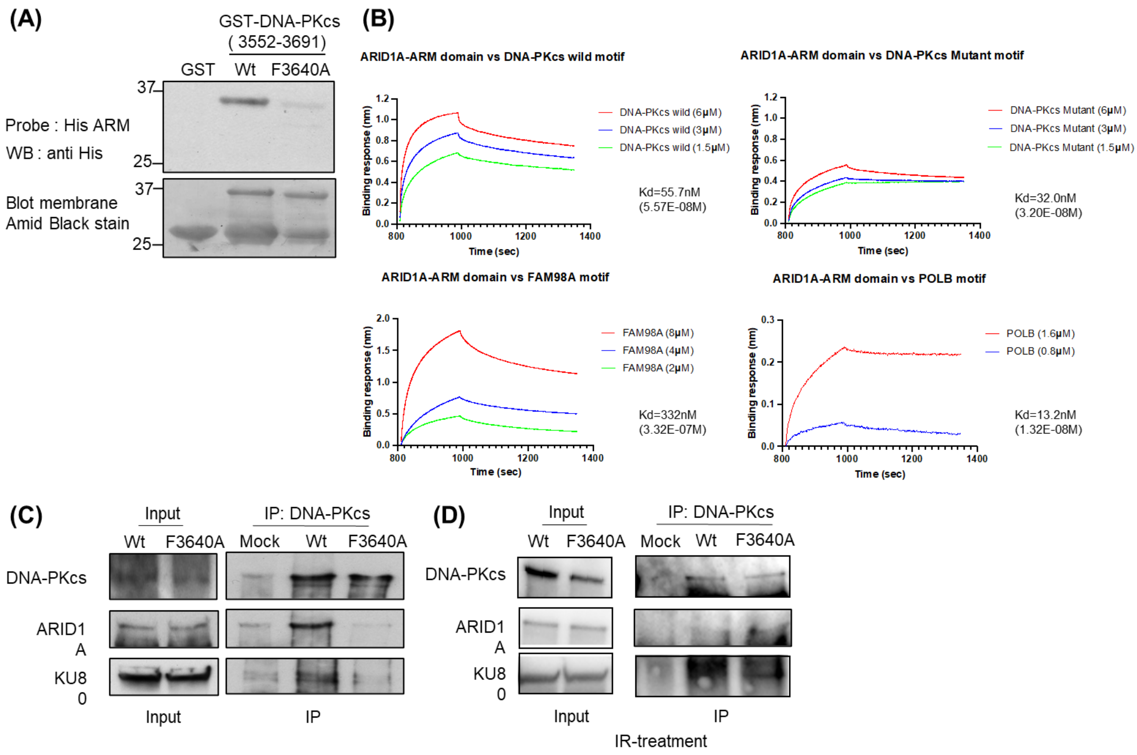

Supplementary Figure S5. ARM domain of ARID1A interacts with DNA-PKcs through its Phe3640 (F3640).

(A) Far-western blotting was used to detect the binding capacity of the wild-type or the F3640A mutant of the GST-DNA-PKcs ARID1A-binding region to the His-tagged ARM domain of ARID1A (aa 3,553–3,691). Moreover, protein staining of the GST-DNA-PKcs-ARID1A-binding domain is shown (lower panel).

(B) Binding kinetics of the His-ARID1A-ARM domain at different concentrations of the Wild-type DNA-PKcs (upper left panel), DNA-PKcs F3640A mutant (upper right panel), FAM98A (lower left panel) and POLB (lower right panel) were measured by BLI binding assay. Calculated dissociation constant (kD) was indicated.

(C) HCT116 wild-type and F3640 mutant cells were harvested and immunoprecipitated

using DNA-PKcs antibody, and immunoblotted with the indicated antibodies.

(D) HCT116 wild-type and F3640 mutant cells were harvested after 10Gy IR-treatment, and immunoprecipitated using DNA-PKcs antibody, and immunoblotted with the indicated antibodies.

Supplementary Figure S6

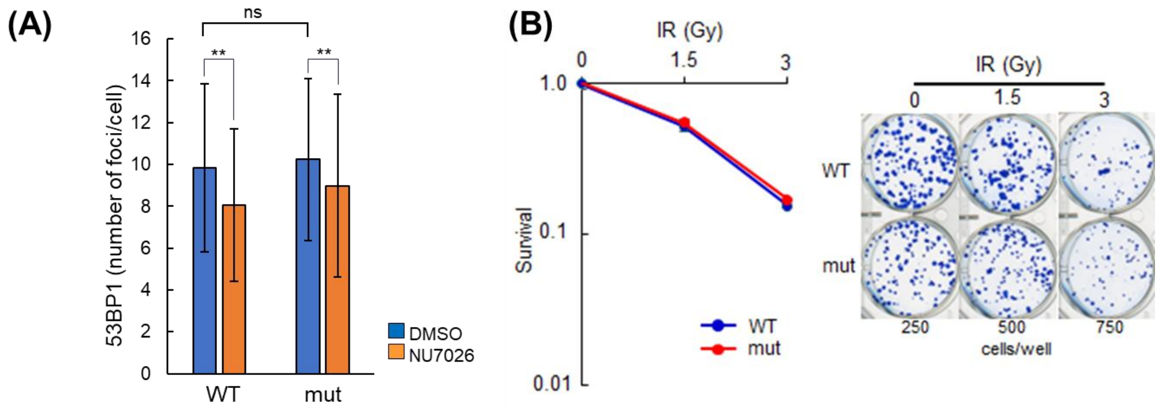

(C)

	G1	G2
Wild type	67.17	13.31
F3640A mutant	66.06	13.84

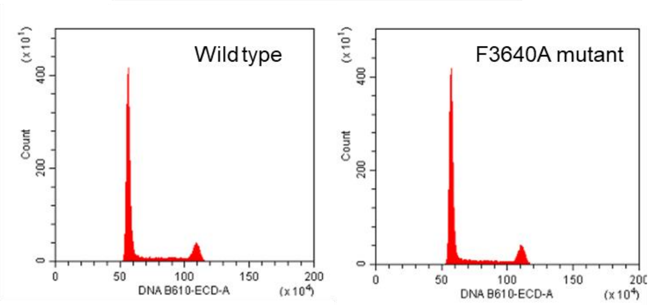

Supplementary Figure S6. F3640 mutation did not affect the colony-forming ability of cells after IR and cell cycle progression.

(A) WT or F3640A knock-in cells (mut) were pretreated with DMSO or NU7026 for 30 min and irradiated with IR (2 Gy). After 15 min, the cells were subjected to immunofluorescent staining with 53BP1 antibody. The number of foci per cell counted by ImageJ is plotted. For each data point, 360–417 cells were analyzed. One of two independent experiments demonstrating similar results is presented. Data are presented as the mean ± SD.

(B) HCT116 wild-type and HCT116 F3640A mutant cells were irradiated at the indicated

doses, and the numbers of colonies were counted 10 days after irradiation.

(C) Flow cytometry was used to analyze cell cycle status in HCT116 wild-type and HCT116 F3640A mutant cells.

Supplementary Figure S7

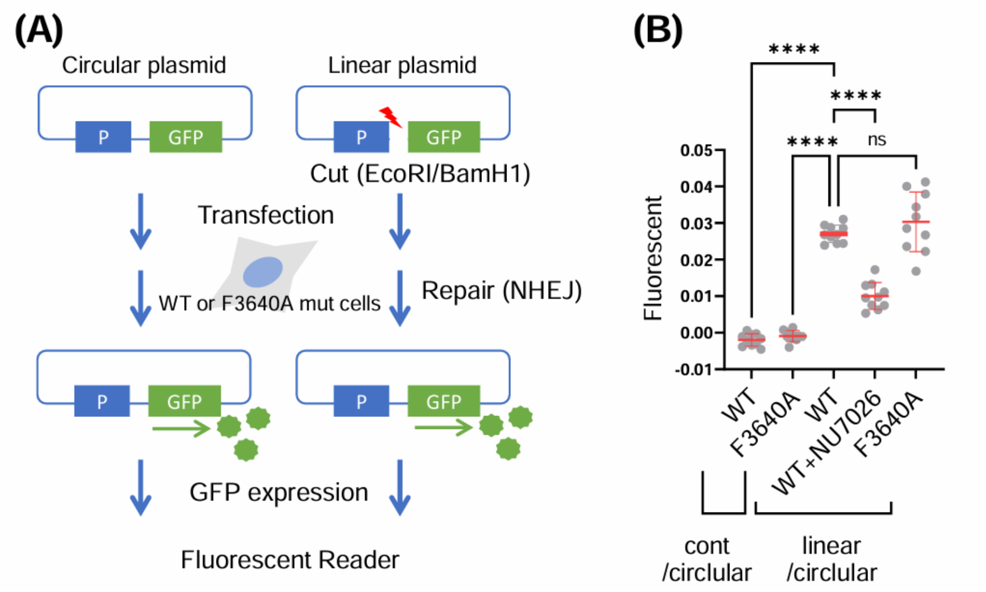

Supplementary Figure S7. F3640 mutation did not affect NHEJ activity.

(A) Scheme of the NHEJ assay using GFP-tagged plasmids is shown; left, circular plasmid transfection; right, linearized plasmid transfection; and P, CMV promoter. After 29 h, intensity of GFP fluorescence were measured by fluorescence reader. Increase in GFP fluorescence transfected with linear plasmids is normalized by GFP fluorescence transfected with circular plasmids.

(B) Increase in GFP fluorescence was measured in HCT116 wild-type (WT) and HCT116 F3640A mutant cells. Left: cont/circular; GFP fluorescence transfected with no plasmid in HCT116 WT or F3640A mutant is normalized by GFP fluorescence transfected with circular plasmids. Right: linear/circular; GFP fluorescence transfected with linearized plasmid in HCT116 WT cells, treated with NU7026 or HCT116 F3640A mutant cells, is normalized by GFP fluorescence transfected with circular plasmids.

Supplementary Figure S8

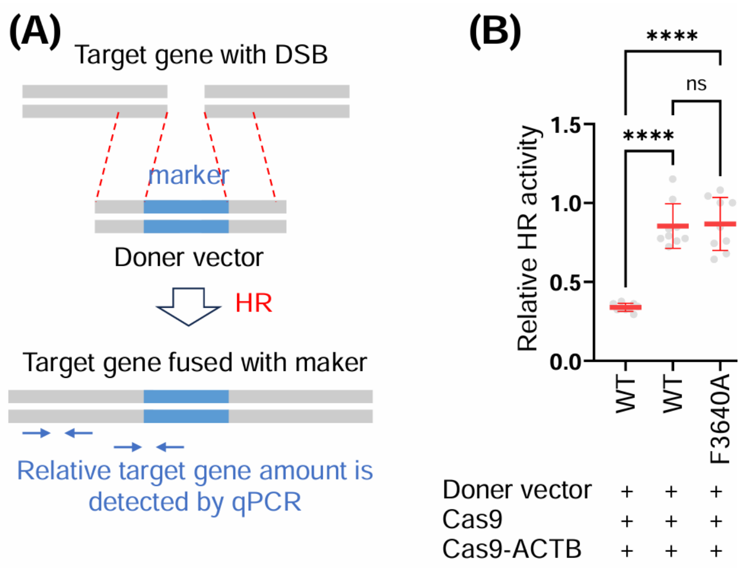

Supplementary Figure S8. F3640 mutation is proficient in HR activity.

Assay for site-specific HR activity was performed in HCT116 wild-type and F3640A mutant cells.

(A) After DSB is induced by ACTB-Cas9, donor vector is targeted in the ACTB sites.

(B) The relative target gene amount is detected by qPCR.

Supplementary Figure S9

(A)

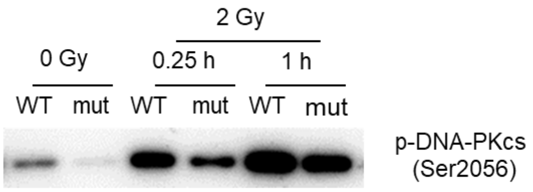

(B)

	0	15 min	15 min +NU7026	30 min	60 min	120 min
WT	297	388	417	354	458	466
F3640A	277	368	360	355	412	435

(C)

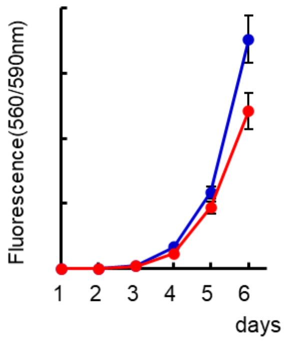

Supplementary Figure S9. F3640 mutation affect autophosphorylation of PCR cluster of DNA-PKcs and cell growth.

(A) Emphasizing the contrast of WB data in Figure 4G reveals that all lanes have signals.

(B) The summary of the number of cells analyzed in Figures 4E, 4F and 4H.

(C) Growth curve of WT and F3640A knock-in cells (mut); cell viability was determined using PrestoBlue Cell Viability Reagents. Data are presented as mean ± SD, n = 3.
